# The development of perceptual averaging: learning what to do, not just how to do it

**DOI:** 10.1111/desc.12584

**Published:** 2017-08-15

**Authors:** Pete R. Jones, Tessa M. Dekker

**Affiliations:** ^1^ Institute of Ophthalmology University College London (UCL) UK; ^2^ NIHR Moorfields Biomedical Research Centre London UK; ^3^ Psychology and Language Sciences University College London (UCL) UK

## Abstract

The mature visual system condenses complex scenes into simple summary statistics (e.g., average size, location, orientation, etc.). However, children, often perform poorly on perceptual averaging tasks. Children's difficulties are typically thought to represent the suboptimal implementation of an adult‐like strategy. This paper examines another possibility: that children actually make decisions in a qualitatively different way to adults (optimal implementation of a non‐ideal strategy).

Ninety children (6–7, 8–9, 10–11 years) and 30 adults were asked to locate the middle of randomly generated dot‐clouds. Nine plausible decision strategies were formulated, and each was fitted to observers' trial‐by‐trial response data (Reverse Correlation). When the number of visual elements was low (*N* < 6), children used a qualitatively different decision strategy from adults: appearing to “join up the dots” and locate the gravitational center of the enclosing shape. Given denser displays, both children and adults used an ideal strategy of arithmetically averaging individual points. Accounting for this difference in decision strategy explained 29% of children's lower precision. These findings suggest that children are not simply suboptimal at performing adult‐like computations, but may at times use sensible, but qualitatively different strategies to make perceptual judgments. Learning which strategy is best in which circumstance might be an important driving factor of perceptual development.

## RESEARCH HIGHLIGHTS

- Children (6–11 years) combined sparse visuospatial information (*N* < 6 elements) in a qualitatively different manner from adults. When asked to “find the middle”, children's responses were best predicted by the gravitational center of a minimum‐bounding shape, whereas adults responded to the arithmetic mean of the observed locations.
- With denser visual displays (*N* > 6 elements), children and adults responded in a qualitatively similar, statistically optimal manner: responding to the arithmetic mean of the visual information.
- Part (~29%) of children's poorer performance in sparse displays was accounted for by their use of a different decision strategy. However, even after accounting for this, children exhibited substantially lower precision than adults. The reasons for this difference remain unaccounted for, but could reflect greater/correlated internal noise, individual differences in decision strategy, or slower learning of task‐relevant information in children.
- Consistent with previous findings, responses were faster when arithmetically averaging displays with more visual elements. This was true for both children and adults, and indicates that summary statistics are subserved by efficient, potentially parallel‐distributed, neural processes.

## 1 INTRODUCTION

**Figure 1:**
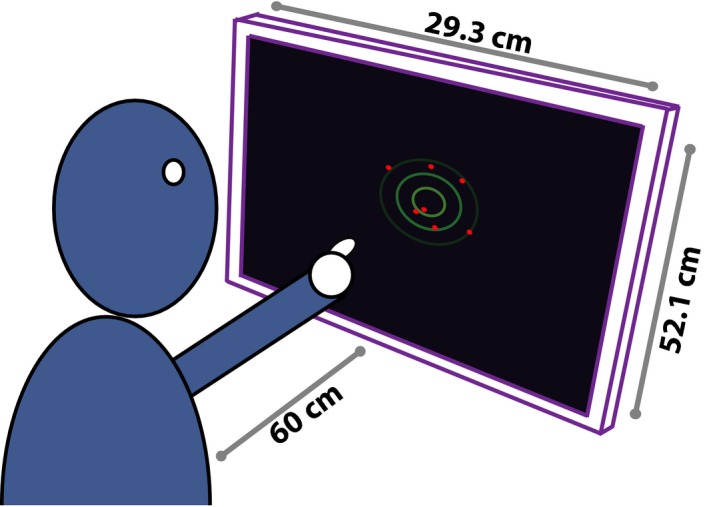
Experimental setup. Observers viewed stimuli consisting of circles (radius 1.1 mm, ~0.11 degrees visual angle), drawn randomly from a symmetric Gaussian distribution (shown here by green isocontours, but not visible to participants). Observers made responses by pressing on an LCD Optical touch‐screen (Prolite T2452MTS; Iiyama Electric Co Ltd, Iiyama, Japan)

The sensory world is stochastic and highly complex. To help separate useful signals from background noise, the mature visual system computes summary statistics that describe central tendencies in the environment (Haberman & Whitney, 2012; Hubert‐Wallander & Boynton, 2015; Peterson & Beach, 1967). For example, when presented with multiple visual objects, adults can rapidly and accurately extract their average location (Alvarez & Oliva, 2008; Badcock, Hess, & Dobbins, 1996; Baud‐Bovy & Soechting, 2001; Bulatov, Bertulis, Gutauskas, Mickiene, & Kadziene, 2010; Hess, Dakin, & Badcock, 1994; Hess & Holliday, 1992; Morgan & Glennerster, 1991; Vos, Bocheva, Yakimoff, & Helspe, 1993; Ward, Casco, & Watt, 1985; Whitaker, McGraw, Pacey, & Barrett, 1996; Whitaker & Walker, 1988) (Figure 1), orientation (Attarha & Moore, 2015; Dakin, Bex, Cass, & Watt, 2009; Dakin & Watt, 1997; Jacoby, Kamke, & Mattingley, 2013; Morgan, Chubb, & Solomon, 2008; Parkes, Lund, Angelucci, Solomon, & Morgan, 2001), size (Ariely, 2001; Chong & Treisman, 2005; Gorea, Belkoura, & Solomon, 2014; Im & Halberda, 2013; Marchant, Simons, & de Fockert, 2013; Price, Kimura, Smith, & Marshall, 2014; Simons & Myczek, 2008; Solomon, Morgan, & Chubb, 2011), brightness (Bauer, 2009), speed (Watamaniuk & Duchon, 1992), or direction of motion (Amano et al., 2012; Burr, 1981; Rocchi, Ledgeway, & Web, 2013; Snowden & Braddick, 1989; Watamaniuk, Sekuler, & Williams, 1989; Williams & Sekuler 1984).^1^

Summary statistics confer two advantages. First, they can improve sensitivity (Alvarez, 2011; Swets, 1959). Noise—both external noise in the environment and internal noise within our own visual system—means that any perceptual estimate of an object's properties is subject to random error. Averaging across multiple estimates causes random errors to cancel out,^2^ resulting in a single overall estimate that is more precise than any individual estimate. Second, summary statistics can reduce key computational demands. Thus, rapidly computing of the “gist” of a scene may help bypass the processing limits imposed by finite memory and attentional resources (Alvarez, 2011; Alvarez & Oliva, 2008; Awh, Barton, & Vogel, 2007; Luck & Vogel, 1997).

While adults are highly adept at computing summary statistics, children appear to struggle. For example, Manning and colleagues (Manning, Dakin, Tibber, & Pellicano, 2014) asked 5–11‐year‐old children to perform a global motion processing task, in which observers were required to estimate the average direction of multiple visual elements, each moving in a slightly different direction. The authors analyzed their data using an *Equivalent Noise* model (Lu & Dosher, 1999). In short, when the amount of external noise (Gaussian direction jitter) was low, performance was assumed to be determined solely by internal noise. When the amount of external noise was high, performance was assumed to be determined solely by under‐sampling. By observing how accuracy declined as external noise increased, the authors inferred that approximately half of the children combined information from more than one element, but that children tended to use fewer elements than adults. Similarly, Sweeny and colleagues (Sweeny, Wurnitsch, Gopnik, & Whitney, 2015) asked 4–5‐year‐old children to perform a size discrimination task, in which the observer must determine which of two arrays was drawn from a distribution with a larger average size (sample discrimination; Jesteadt, Nizami, & Schairer, 2003). Following Bernoulli's theorem (Feller, 1968), as the number of samples increases, response accuracy should improve, at a rate of $\sqrt{N}$. Accordingly, children became more accurate as the number of samples increased, but the improvement was smaller than for adults, or than would be predicted by an ideal observer.

In short, both studies observed suboptimal integration in children, and in both cases the authors attributed this to children ignoring some of the available information, perhaps due to immaturities in selective attention (Jones, Moore, & Amitay, 2015; Ristic & Kingstone, 2009) or memory (Cowan, AuBuchon, Gilchrist, Ricker, & Saults, 2011; Simmering, 2012). Effectively, they suggested that children attempted to respond in the same way as adults (mean‐averaging the information presented), but that their implementation was imperfect. In this case, development can be seen as a form of parametric learning, that is, learning the optimal values of the various “variables” involved in a computational process—such as the optimal “weight” to give each source of information (e.g., see Equation 3). In machine learning terms, children are struggling with a problem of *optimization*.

However, there exists a second class of explanation that also fits the existing data: it may be that children use qualitatively different strategies to summarize sensory information. For example, in the case of size judgments, instead of arithmetically averaging independent estimates of sizes, children may be responding based on the total surface area of the display, the size of the largest single element, the density of the elements, or so forth. These strategies may not be what the experimenter intended/expected when they designed their task, and may be suboptimal given the demands of the current task. But nonetheless, such strategies are often quite rational, and enable the participant to operate at a better‐than‐chance level. In this case, development can be seen as a form of structural learning (Wolpert & Flanagan, 2010), that is, learning what is the best overall “equation” to solve the task in the first place, including what the sources of information are, and how best to map these sensory inputs to a final decision. In machine learning terms, children are struggling with a problem of *model selection*.

That observers may employ different strategies to solve basic psychophysical tasks has been known for as long as experimental psychology has been practiced. Indeed, Sweeney et al. (2015) explicitly controlled for two such explanations by equating each array of cues for total surface area and density. However, this does not rule out an infinite number of *other* response strategies. Nor, by treating alternative response strategies as confounds to be controlled for, are we able to assess how important this potential source of inefficiency may be in children's everyday lives.

Previous studies are unable to distinguish between these two classes of explanation (parametric vs. structural learning) because they rely on traditional, “molar” measures, such as *d′*, percent correct, or psychophysical threshold. Such measures are computed by averaging response‐data across multiple trials. They show *how well* observers are performing, but provide no insights into *why* performance may vary. To discriminate between parametric and structural hypotheses, the present study therefore employed a trial‐by‐trial (“molecular”; Berg, 2004) method of analysis, designed to reveal systematic differences in how children and adults average visual stimuli.

**Figure 2:**
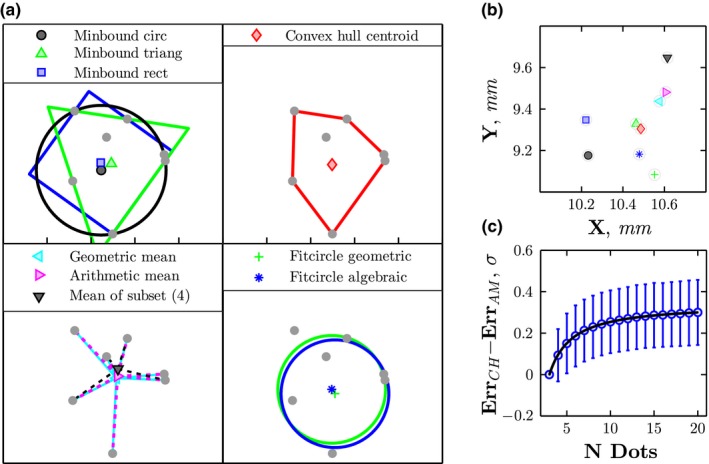
(a) The nine possible decision strategies for integrating spatial information considered in the present study. See Table I for text descriptions. (b) The predictions of each strategy, for a single representative trial (raw data not shown). (c) The mean Euclidean difference (± 1 *SD*) between the predictions of the convex hull and arithmetic mean strategies, as a function of the number of data points, determined using 20,000 simulated trials. Note that when *N* = 3 (fewest *N Dots tested)*, the predicted responses for both strategies are identical—becoming increasingly distinct as *N* increases

In the present study, children (6–11 years) and adults were asked to “find the middle” of a cloud of dots sampled from a 2D Gaussian distribution (Figure 1; Gaussian‐jittered spatial information). Crucially, unlike traditional methods of analysis, we did not score responses as “correct” or “incorrect”. Instead, we formulated a range of plausible algorithms that observers might employ, and determined which of these best predicted the empirical, trial‐by‐trial response‐data (irrespective of whether these responses were accurate or not). Perhaps surprisingly, a large number of strategies can be devised to perform this simple spatial averaging task (Figure 2a). For example, an observer might (i) mean‐average the Cartesian coordinates of each individual dot, (ii) fit a shape to the dot cloud and locate its center of gravity, or (iii) try to determine the smallest surface that would enclose the observed dots and locate the center of that (see Table 1). Notably, each of these strategies predicts a quantitatively different response (Figure 2b, though cf. Figure 2c). The difference between observed and predicted behavior on each trial can therefore be used to determine the best‐fitting model of decision‐making.

**Table 1:** The nine possible decision strategies that were considered in the present study. See Figure 2 for graphic examples of each

Name	Description
Minbound circ	Centroid of the minimum radius bounding circle (the smallest circle that encloses all *<x,y>* points)
Minbound triang	Centroid of the minimum area bounding triangle
Minbound rect	Centroid of the minimum area bounding rectangle
Convex hull	Centroid of Convex hull (the smallest convex polgyon that encloses all *<x,y>* points)
Geometric mean	The geometric mean of all *<x,y>* points
Arithmetic mean	The arithmetic mean of all *<x,y>* points
Mean of subset (*N*)	The arithmetic mean of at most *N <x,y>* points, excluding the most distant outliers
Fitcircle geometric	Centroid of the best fitting circle, minimizing residual geometric error (sum of squared distances between the observed *<x,y>* points and the fitted circle), fitted using nonlinear least squares (Gauss Newton).
Fitcircle algebraic	Centroid of the best fitting circle, minimizing residual error (sum of distances between the observed *<x,y>* points and the fitted circle), fitted algebraically.

Participants received points for hitting a target centered on the arithmetic mean of the underlying distribution. Given this reward‐structure, the statistically optimal (“maximum likelihood”) strategy is to compute the arithmetic mean of the observed locations. Based on previous data (Morgan & Glennerster, 1991), we predicted that adults would respond in this way. In contrast, it was unknown how children would behave. If children use suboptimal decision strategies (structural inefficiency), then we would expect to see systematic differences in their preferred decision strategy. Alternatively, children may average spatial information in a qualitatively similar manner to adults, but may only attend to a subset of the information available (i.e., fail to “weight” every cue appropriately—parametric inefficiency). In this case, we would predict no systematic differences in preferred decision strategy but only an increase in response variability.

## 2 METHODS

### 2.1 Participants

Ninety children (6–7, 8–9, 10–11 years; *N* = 30 per group) and 30 adults (19–42 years; μ = 24 years) participated. Written consent was obtained from all participants (adults), or from a responsible caregiver (children), and children gave verbal assent to participate. The study was conducted in accordance with UCL Research Ethics Committee approval (#2280/001). Participants were reimbursed for their time (£7.50 for adults, small toys for children).

### 2.2 Stimuli and procedure

Participants were asked to find the middle of a cloud of dots (see Figure 1), using a stimulus design developed and described previously by Juni and colleagues (Juni, Gureckis, & Maloney, 2015). Each dot was an anti‐aliased circle, 1.1 mm in radius (~0.11 degrees visual angle), generated in Matlab (Mathworks, Natick, USA) using Psychtoolbox‐3 (Brainard, 1997), and presented on an LCD optical touch‐screen (Prolite T2452MTS; Iiyama Electric Co. Ltd, Iiyama, Japan). On each trial, *N* dots (see below) were randomly sampled from a randomly located symmetric bivariate Gaussian distribution, and participants were asked to locate the middle of the dots by pressing on the screen. The location of each dot was therefore a noisy (Gaussian jittered) but unbiased estimate of the center of the underlying distribution. The center of the underlying distribution varied randomly between trials, and was constrained such that 98% of the distribution fell inside the visible screen area. Participants were given feedback after each trial: scored correct if within 12.8 mm of the arithmetic mean of the underlying sampling distribution^3^. The ideal strategy was therefore to respond to the arithmetic mean of the observed dots (see Juni et al. 2015). For 60 participants (*N* = 45 children) the standard deviation of the bivariate Gaussian sampling distribution, σ_xy_, was 12.5 mm. For the other 60 participants, σ_xy_ = 27.5 mm. This difference was not pertinent to the present study (NB: the data reported here formed part of a wider dataset, additional data from which are reported elsewhere; Jones et al., under review), and the results did not differ qualitatively between the two conditions (NB: participants *did* tend to be less accurate in noisier conditions, but did not appear to differ in terms of their preferred response strategy). Data from both conditions were therefore analyzed together. Participants completed approximately 150 trials on average (the exact number of trials varied between participants: μ = 154,, σ = 34). Trials were randomly distributed across six *N Dot* conditions: 〈3, 4, 5, 6, 7, 15〉, for a total of approximately 4500 trials per age group.

### 2.3 Potential observer strategies for locating the “middle” of a cloud of dots

Nine potential decision strategies were considered.^4^ These are shown graphically in Figure 2a, and are detailed in Table 1. The set of strategies was not exhaustive, but included all plausible strategies that we were able to devise, and was representative of the types of strategies observers anecdotally reported using when questioned.

#### 2.3.1 Centroid of a minimum bounding polygon

In the minimum‐bound approaches (Table 1; R1–R3), the observer visualizes the smallest shape (circle, triangle, or rectangle) that encloses the set of observed points, and then computes the centroid of this shape. Here we define “smallest” shape as the shape with least area. Smallest could also be defined in terms of perimeter. However, minimizing area of perimeter yields identical predictions in most cases, and negligible differences in the remaining cases. The centroid (“geometric center”) of a two‐dimensional region is the arithmetic mean position of all the points in the shape, which can be computed from the vertices of the minimal bounding polygon as follows:

(1) $$\begin{array}{cc} & C_{x}=\frac{1}{6A}\sum_{i=1}^{n}\left(x_{i}+x_{i+1}\right)\left(x_{i}y_{i+1}-x_{i+1}y_{i}\right)\\ & C_{y}=\frac{1}{6A}\sum_{i=1}^{n}\left(y_{i}+y_{i+1}\right)\left(x_{i}y_{i+1}-x_{i+1}y_{i}\right) \end{array}$$

where (*x*_1_,*y*_1_), (*x*_2_,*y*_2_), …, (*x*_*n*_,*y*_*n*_) are the vertices of the *n*‐sided polygon, arranged in clockwise order around the perimeter and with the first vertex repeated at the end to close the shape (i.e., x_1_ = x_n+1_, and y_1_ = y_n+1_). The variable *A* is the signed area of the polygon:

(2) $$A=\frac{1}{2}\sum_{i=1}^{n}\left(x_{i}y_{i+1}-x_{i+1}y_{i}\right)$$

#### 2.3.2 Centroid of a convex hull

The convex hull is the smallest polygon that encloses the set of observed points. It can be thought of a rubber band around all of the observed points. It is a generalization of the “minimum bound” approaches described above. However, with a convex hull strategy the observer is not constrained to visualize a shape of any particular geometric form.

#### 2.3.3 Arithmetic mean

The arithmetic mean was defined as per usual, and was computed independently for the *x* and *y* coordinates. As detailed in the introduction, this is the ideal strategy for computing the central tendency of independent observations of a random variable, and it was the expected strategy of adults (Morgan & Glennerster, 1991).

(3) $$\sum_{i=1}^{N}\omega_{i}x_{i}\;\text{where}\;\omega_{i}=\frac{1}{N}$$

#### 2.3.4 Mean of subset (*N*)

This was the same as the arithmetic mean, but was based on a subset of *N* dots. When the number of dots presented was greater than *N*, the most *outlying* dots were excluded. In practice this is only one of an infinite number of ways to partition the data, but ignoring outliers seemed a reasonable approximation for how an observer might pick out a subset of points. Outliers were determined by the median distance between each point and every other point (Rousseux & Croux's “S_n_ factor”; Rousseeuw & Croux, 1993). In the reported data, *N* was set to four, as this was the number of samples that Sweeny and colleagues reported children use when performing size‐averaging (Sweeny et al., 2015). Other values of *N* (3, 5, 6, 7) were also analyzed, but these results are not reported as they were qualitatively identical to *N* = 4.

#### 2.3.5 Geometric mean

The geometric mean is analogous to the arithmetic mean, but uses multiplications and root instead of additions and division. It is equivalent to the arithmetic mean of the logarithm‐transformed location values (with the product then returned to the original, unlogged scale). The geometric mean might be appropriate, therefore, if spatial information in the brain is distributed along a logarithmic decision axis.

(4) $${\left(\prod_{i=1}^{N}x_{i}\right)}^{1/N}$$

#### 2.3.6 Centroid of a fitted circle

In the fitted circle approach, the observer mentally fits a circle to the observed points, and responds to its centroid. Unlike the minimum‐bounding approaches, the circle passes through the points, rather than enclosing them. One way to fit such a circle is to algebraically specify a circle in a plane, and then determine analytically the coefficients *a*,* b*, and *c*, that provide the best linear fit to the data (see Gander, Golub, & Strebel, 1994):

(5) $$F\left(x\right)=ax^{T}x+b^{T}x+c=0$$

An alternative (“geometric”) approach uses an iterative algorithm to minimize the sum of the squared distances from the circle to the observed points. As discussed by previous authors (Gander et al., 1994), geometric fitting often provides different results from algebraic fitting, and is liable to produce fits that are in greater accord with our intuitions.

### 2.4 Analysis

All analyses were performed using the data from all participants within each age group. Concatenating data across observers was necessary to constrain the models adequately, given the relatively small amount of trials per participant (μ = 25, per *N‐*dots condition). However, it meant that we could not examine individual differences in decision strategies (Haberman, Brady, & Alvarez, 2015). Responses time data were log_10_ transformed prior to statistical analyses to ensure normality.

### 2.5 Evaluating observer strategies

To evaluate how well each strategy predicted the observer's behavior we computed mean error: the mean Euclidean distance between the predicted and observed response for each trial (i.e., the residual error). More predictive strategies should exhibit lower mean error, and for the ideal observer mean error is zero. Non‐parametric bootstrapping was used to compute 95% confidence intervals around mean error values.

Subjects may not rely on one single perceptual averaging strategy, but may instead shift between two or more depending on the stimulus condition. We quantified this by computing a relative decision weight for each strategy using the reverse correlation method (Lutfi, 1995; Richards & Zhu, 1994). In brief, a multiple multivariate linear regression was performed, containing: (i) x and y error terms, (ii) two independent variables per strategy 〈x_predicted_, y_predicted_〉, and (iii) two dependent variables 〈x_observed_, y_observed_〉. The x and y slope coefficients were then averaged within each strategy, and normalized so that their magnitudes summed to one. This yielded one relative weight value, ω, per strategy, indicating the relative degree to which that strategy determined the observer's responses. More predictive strategies exhibit higher weight values, with the maximum being ω = 1.

### 2.6 Characterizing performance

To investigate the effect of preferred summarizing strategy on performance, we computed traditional measures of precision, accuracy, and response latency.

#### 2.6.1 Precision

Response precision was quantified as the reciprocal of standard distance deviation (1/*SDD*). *SDD* is the two‐dimensional equivalent of standard deviation, and is computed as:

(6) $$SDD=\sqrt{\sum_{i=1}^{N}\frac{d_{i}^{2}}{N-2}}$$

where *N* is the total number of trials, and *d*_*i*_ is the residual error on trial *i* (the distance between predicted and observed response, in millimetres):

(7) $$d=\sqrt{{\left(R_{x}-\overset{¯}{x}\right)}^{2}+{\left(R_{y}-\overset{¯}{y}\right)}^{2}}$$

where <R_x_,R_y_> are the participant's response coordinates, and $\left\langle \overset{¯}{x},\overset{¯}{y}\right\rangle$ are the arithmetic mean of the observed dots. Note that implicit in this formula is an assumed decision strategy, since errors are computed relative to the arithmetic mean of the observed points. This is problematic, since an ideal observer who does not respond to the arithmetic mean of the data will exhibit *SDD* > 0 (despite, by definition, having infinitely high precision). Alternatively then, distance can be computed by replacing the terms $\left\langle \overset{¯}{x},\overset{¯}{y}\right\rangle$ with the target coordinates predicted by a different, more appropriate decision strategy. For example, given an observer who computes the geometric mean of the observed data, the appropriate measure of residual error can be derived by combining Equations 4 and 7, thus:

(8) $$d=\sqrt{{\left(R_{x}-{\left(\prod_{i=1}^{N}x_{i}\right)}^{1/N}\right)}^{2}+{\left(R_{y}-{\left(\prod_{i=1}^{N}y_{i}\right)}^{1/N}\right)}^{2}}$$

In the present analysis, we therefore began by using the simple (“traditional”) measure of response error given Equation 7, but went on to consider more appropriate measures, given observers' empirically estimated decision strategies.

#### 2.6.2 Accuracy

Response bias (accuracy) was quantified as the mean signed deviation of responses to the arithmetic mean of observed points:

(9) $$\beta_{xy}=\frac{\frac{1}{N}\sum_{i=1}^{N}\left(R_{x}-\overset{¯}{x}\right)+\frac{1}{N}\sum_{i=1}^{N}\left(R_{y}-\overset{¯}{y}\right)}{\sqrt{2}}$$

In an unbiased observer, β_xy_ = 0.

#### 2.6.3 Response latency

Response latency was quantified as the lag between stimulus presentation and the observer's response, in seconds. Note, however, that responses were not speeded, and participants were instructed only to be as accurate as possible. To ensure statistical normality, reaction time data were log‐transformed prior to analysis (Whelan, 2008).

### 2.7 Analyzing age differences using bootstrapping

To evaluate differences in *SDD* between different age groups (e.g., 6–7‐year‐olds vs. adult controls) a bootstrapping procedure was used. Samples were randomly drawn, with replacement, from each of the two age groups, and the difference in mean *SDD* was computed. This procedure was repeated 20,000 times. The *p*‐value was defined as 2*P*, where *P* was the proportion of these 20,000 differences that had the opposite sign from the observed difference in *SDD*. This procedure is fundamentally similar to performing traditional hypothesis test (*t* test, Mann‐Whitney U test), or to graphically comparing bootstrapped confidence intervals.

## 3 RESULTS

**Figure 3:**
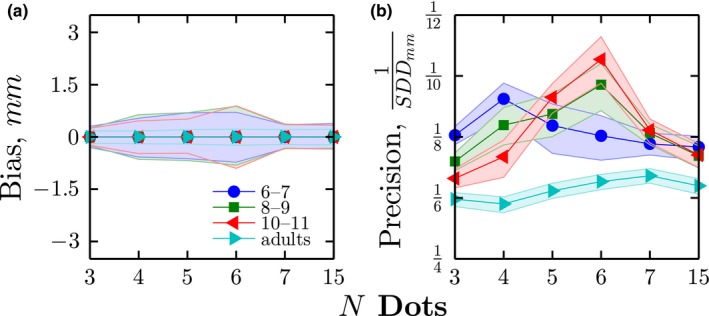
(a) Mean (± 95% CI) response bias (Eq 4) for each age group. (b) Mean (± 95% CI) response precision (see Eq 6), for each age group. Note that this is computed with respect to an ideal strategy of arithmetic mean computation. Error bars were computed using bootstrapping (N = 20,000)

To characterize overall performance, precision (1/*SDD*) and bias (β_xy_) were computed for each age group. As shown in Figure 3, no significant response bias was present in any group (Figure 3a), but precision was significantly lower for children than adults (Figure 3b). The difference in precision between children and adults was confirmed by using bootstrapping to perform group comparisons, as described in the Methods (*p* < .01 for Children vs. Adults, for all combinations of *Age Group* × *N Dots*).

**Figure 4:**
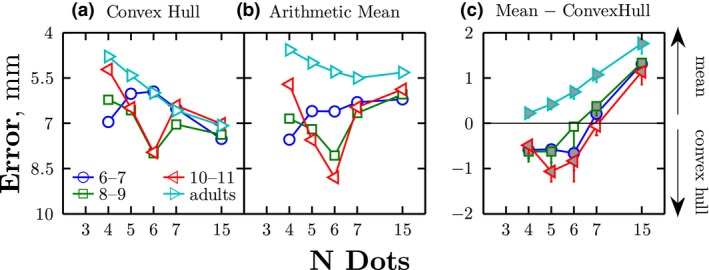
Reliance on convex hull versus arithmetic mean decision strategies, as assessed using relative weights. Other strategies were also included in the model, but are not shown here as they were generally given close to zero weight. (a–b) Individual weights for each individual strategy: higher ω indicates more predictive of observed behavior. (c) Direct comparison between the two strategies (Δ_ω_ = ω_mean_ – ω_cvxhull_). Values greater than zero indicating that observers' responses were better predicted by the arithmetic mean strategy. Values less than zero indicating that observers' responses were better predicted by the convex hull strategy. Error bars indicate 95% Confidence Intervals, derived using bootstrapping (*N* = 20,000). Values significantly different from zero have been shaded grey. These data essentially confirm graphically what can also be seen in Table 2, using a different method of analysis

To investigate which decision strategy children and adults used to perform the task, each of the nine algorithms shown in Figure 2 was fitted to the trial‐by‐trial response data from each age group, and mean error computed. As shown in Table 2, adults responded based on the arithmetic mean of the observed data, irrespective of number of visual elements. By contrast, children of all ages only computed the arithmetic mean when the number of dots was large (*N* = 15). When there were fewer dots, children appeared to mentally draw a shape around the dots, and responded to its centroid. In the case of *N* = 3, this shape tended to be a circle. With intermediate numbers of dots (*N* = 4 or 5) children drew a more general convex hull. Children's change in decision strategy with number of elements is shown graphically in Figure 4. By inspection, it can be seen that the transition in strategy occurred in children at approximately *N* = 6–7 elements. This change was confirmed statistically using bootstrapping (Figure 4, shaded markers). Thus, with children of all ages, their responses were significantly better predicted by the convex hull centroid when *N* = 4 or 5 (all *p* < .01), and by arithmetic mean averaging when *N* = 15 (all *p* < .01). At intermediate numbers of dots (*N* = 6 or 7) there was no clear pattern, with some age groups showing no significant preference for one strategy or the other. Note, crucially, that even if noise was added to the arithmetic mean model, this could not, by definition, improve its predictive power. These data therefore demonstrate unambiguously that children are not simply computing the mean‐average position (i.e., but noisily), when *N* ≲ 6. Other strategies (e.g., geometric mean, averaging a subset, minimum bounding square, best‐fitting circle, etc.) gave poor accounts of how children or adults responded in any condition (see Table 2).

**Table 2:** Mean Euclidean error between observed responses and the predicted responses given each of eight putative decision strategies (see Supplemental Material for analogous assessments based on relative weights and percent best). Bold figures indicate the best fitting model for each *N* Dots condition. The difference between the arithmetic mean and convex hull strategies is also shown graphically in Figure 4

Mean Error, *mm*	N Dots					
	3	4	5	6	7	15
6‐7 years						
mindbound circ	**6.0**	8.2	7.9	8.1	9.2	10.1
minbound triang	6.6	7.8	6.9	6.8	7.3	8.9
minbound rect	6.7	8.9	7.9	8.0	8.7	9.1
convex hull	6.6	**7.0**	**6.0**	**6.0**	6.5	7.5
geometric mean	6.7	7.6	6.7	6.9	6.6	6.4
arithmetic mean	6.6	7.5	6.6	6.6	**6.3**	**6.2**
mean of subset (4)	6.6	7.5	10.9	12.3	11.7	10.0
non‐lin fit circ	19.3	16.6	15.8	16.1	17.7	13.5
linear fit circ	19.3	15.2	11.4	10.3	10.4	9.3
8‐9 years						
mindbound circ	**5.7**	6.9	7.8	10.4	9.8	10.1
minbound triang	5.7	7.1	7.6	8.7	7.9	8.8
minbound rect	6.3	7.8	8.2	10.0	9.0	9.2
convex hull	5.7	**6.2**	**6.6**	**8.0**	7.0	7.4
geometric mean	5.9	7.0	7.5	8.3	6.8	6.4
arithmetic mean	5.7	6.8	7.2	8.1	**6.7**	**6.0**
mean of subset **(4)**	5.7	6.8	12.9	13.3	11.8	9.8
non‐lin fit circ	17.1	14.4	15.4	18.8	17.6	14.5
linear fit circ	17.1	11.4	10.7	12.3	11.3	9.5
10‐11 years						
mindbound circ	**4.9**	6.0	8.0	10.0	8.8	9.9
minbound triang	5.1	6.1	7.7	9.3	7.7	8.2
minbound rect	5.7	6.6	8.5	10.4	8.7	9.0
convex hull	5.1	**5.2**	**6.5**	**8.0**	**6.4**	7.0
geometric mean	5.3	5.9	7.7	8.9	6.6	6.1
arithmetic mean	5.1	5.7	7.6	8.8	6.5	**5.9**
mean of subset **(4)**	5.1	5.7	13.7	16.9	12.8	9.8
non‐lin fit circ	17.4	15.8	16.7	17.2	17.6	13.8
linear fit circ	17.4	11.0	11.6	12.0	10.8	9.4
Adults						
mindbound circ	6.0	6.7	7.6	8.7	9.4	9.9
minbound triang	4.7	5.1	5.9	6.8	7.5	8.4
minbound rect	6.3	6.6	7.3	8.3	8.7	8.8
convex hull	4.7	4.8	5.4	6.0	6.6	7.1
geometric mean	5.0	4.9	5.3	5.7	5.8	5.7
arithmetic mean	**4.7**	**4.6**	**5.0**	**5.3**	**5.5**	**5.3**
mean of subset (4)	**4.7**	**4.6**	9.4	10.7	10.6	9.0
non‐lin fit circ	18.5	17.4	17.2	18.0	17.9	14.2
linear fit circ	18.5	13.4	11.5	11.6	11.5	9.3

**Figure 5:**
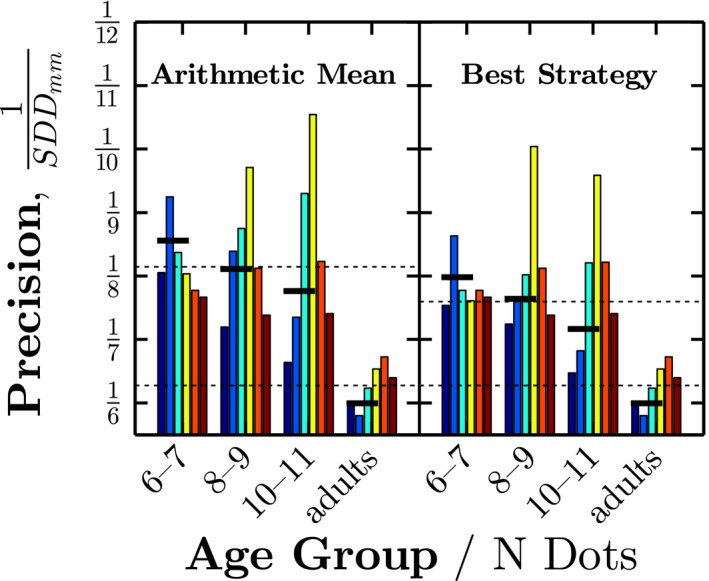
Response precision, when (*left*) residual error is computed based on the arithmetic mean of the data, and (*right*) when residual error is computed using the best‐fitting decision strategy for that age group / dot condition (as shown in Table 2). See Methods 2.6 for further details. Bold horizontal lines show mean precision for the *N* = 3–6 conditions (i.e., where children's strategies differed from adults'). Dashed horizontal lines show mean precision for all dot conditions, averaged within adults and children. By comparing between panels, one can see how much of the difference in response variability (% *SDD*) between children and adults was explained by differences in decision strategy

The foregoing indicated that children differed from adults in how they localized small numbers of dots (*N* ≲ 6). To what extent can this qualitative difference in strategy explain children's failure to average information efficiently? That is, to what extent can differences in strategy explain the difference in localization precision between children and adults shown previously in Figure 3b? To address this question, response precision (1/*SDD*) was recomputed for each condition, using the expected $\left\langle \overset{¯}{x},\overset{¯}{y}\right\rangle$ values for the best fitting strategy for that age‐group/dot‐condition. Once these adjustments for decision strategy were performed, observed precision within children improved substantially (Figure 5). For example, in the *N* = 3–5 conditions, use of a different strategy accounted on average for 29% of the apparent difference between children and adults.^5^ This indicates that some, but not all, of children's immaturities are due to their use of qualitatively different decision strategies.

**Figure 6:**
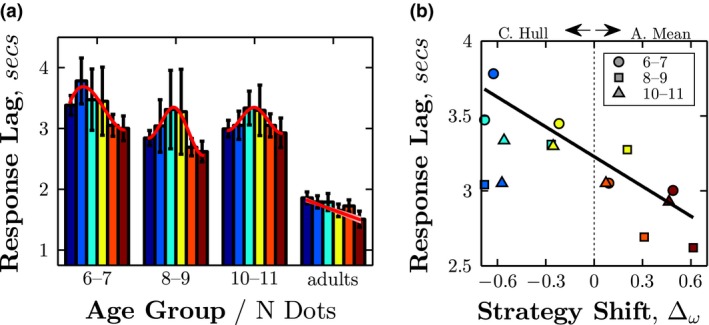
Response time data. (a) Median (± 95% CI) response times, as function of Age Group and condition (*N Dots*). Confidence intervals were computed using bootstrapping (*N* = 20,000). (b) Association between the change in strategy from convex hull to arithmetic mean (i.e., the data points from the rightmost panel of Figure 4), and median response time. A higher value of Δω indicates greater reliance on the arithmetic mean strategy. The line indicates the least‐square geometric mean (“reduced major axis”) linear regression fit

To further examine age differences in decision strategy with different *N dots*, response latency was also analyzed (although note that responses were not speeded, and participants were instructed only to be as accurate as possible). The results are shown in Figure 6. Adults were on average faster to respond than children (independent *t* test comparison of log_10_ data; *p* ≪ .001, for all three Age Group comparisons). More importantly, the pattern of response latency varied between children and adults. In adults, median response time decreased linearly with *N Dots* (least‐squares linear regression; *F*_4_ = 28.66, *p* = .006, *r* ^2^ = 0.88), indicating that they took less time to average more visual elements. By contrast, children's response times *increased* for small numbers of dots, but *decreased* for larger *N* (Figure 6, red curves), and these variations in log‐transformed response times correlated strongly with the changes in decision strategy shown previously in Figure 4 (Figure 6b; *r*_13_ = −0.66, *p* = .007). In particular, a post hoc test (between‐subjects ANOVA of *N* dots (two levels: 5, 7) versus Age Group (three levels: 6–7, 8–9, 10–11)) found that children's response times decreased significantly between *N* = 5 and *N* = 7 dots across all age groups (Main effect of *N* Dots: F(1, 4492) = 31.61, p < .001, ηp^2^ = 0.01; No interaction effect between *N* Dots and Age Group: F(2, 4492) = 1.15, p = .318, *ns*). Thus, children's responses became faster around the point at which they appeared to switch between convex hull and arithmetic mean averaging strategies (see Figure 4c).

## 4 DISCUSSION

Adults, and children aged 6 to 11 years were able to combine visuospatial information, in order to locate the center of a set of elements. Children exhibited higher response variance (lower precision) than adults. This was shown to partially reflect structural learning: children used a qualitatively different strategy from adults when the number of dots was low, opting to “join the dots” and respond to the center of the smallest geometric shape that enclosed the observed points (decision‐strategy 4, Table 2). Such strategies may be perfectly sufficient in some contexts, but were suboptimal for the present task. In contrast, adults responded in the statistically optimal manner, by computing the arithmetic mean of the elements; and they did so consistently, irrespective of the number of elements. Accounting for this difference in strategy explained 29% of the difference in precision between children and adults. This finding is analogous to a recent study in audition, which showed that developmental difference in the slope of a psychometric functions can be explained by taking into account differences in decision strategy (Jones et al., 2015).

In the past, poorer performance on perceptual‐averaging tasks has been attributed solely to the inefficient implementation of an ideal strategy (parametric learning). For example, Sweeny et al. (2015) interpreted immature size‐averaging as indicating that children pool information across fewer objects than adults. Similarly, Manning et al. (2014) interpreted immature motion‐averaging as indicating that older children “are able to [effectively] average across more local motion estimates”. The present study expands upon this prior work by showing that children are additionally limited by their use of altogether different response strategies. In this light, development may take place at a qualitatively different level of decision‐making than previously proposed: namely through children learning how to identify the “form” of a task and how best to approach the problem (structural learning), rather than by learning how best to optimize their implementation of a particular solution (parametric learning).

Of course, these two types of development are not mutually exclusive, and even in the present work much (71%) of children's increased error remained unaccounted for. This may indicate that children are further limited by how efficiently they are able to implement their preferred strategy, as has been suggested previously by others (Manning et al., 2014; Sweeny et al., 2015) (i.e., they might under‐ or over‐”weight” some of the available cues). Alternatively/additionally, it may be that children are limited by sources of *random* inefficiency (“internal noise”), either at the sensory level, or in terms of high‐level inattentiveness. Given the nature of the present task, internal noise at the motor level may also have limited children's ability to respond precisely. Finally, it may be that some of the remaining developmental difference can be explained by *deterministic* factors that we were unable to examine in the present work, such as how response strategies differ across individuals, or vary with practice (Jones, Moore, Shub, & Amitay, 2014; i.e., with children being slower to learn the ideal response strategy). With the present data, we are unable to test these various hypotheses, and in future it would be interesting to collect larger and more nuanced datasets that could do so. For example, one could repeat certain stimuli configurations throughout the course of the experiment, and use the inconsistency of observers' responses as an index of internal noise (Green, 1964; Jones, Shub, Moore, & Amitay, 2013). Notably though, none of these explanations can explain the systematic differences in responses that were observed in the present study (e.g., no source of random error would cause one response strategy to consistently predict observers' responses more accurately than another). Accordingly, the present findings show unambiguously that at least *part* (29%) of the difference in performance between adults and children is explained by the use of qualitative different algorithms (structural learning) rather than quantitative differences in response efficiency (parametric learning).

Perhaps surprisingly, children switched to using the ideal, adult‐like decision strategy when the number of dots was high (*N* ≳ 6). This demonstrates that children are liable to vary their decision strategy depending on the stimulus parameters. In this respect, it is interesting to note that many studies that have reported immaturities in cue integration have tended to use very small numbers of cues (Gori, Del Viva, Sandini, & Burr, 2008; Nardini, Jones, Bedford, & Braddick, 2008; Petrini, Jones, Smith, & Nardini, 2015; Sweeny et al., 2015). In the present work, children actually performed relatively poorly with such sparse inputs, and became faster and more accurate when viewing more complex stimuli, presumably because with increasing complexity, non‐ideal strategies became increasingly costly. One corollary of this is that at times, by simplifying psychophysical experiments for children, we may actually be underestimating their perceptual abilities.

Children's change in decision strategy was corroborated by their response time data, with children responding *faster* as they switched from a convex hull to an arithmetic averaging strategy (i.e., as the number of dots increased). The counter‐intuitive finding that responses actually became faster as the visual scene became more complex is consistent with similar previous findings in adults (Robitaille & Harris, 2011), and supports the notion that summary statistics represent, in part, a computationally expedient mechanism for perceptual decision‐making (Haberman & Whitney, 2012). Our findings suggest that it may take the developing system many years to identify the most expedient summarizing strategy, but that even young children are capable of utilizing adult‐like strategies when stimulus complexity makes the use of more simple heuristics untenable.

In the present study, structural learning (i.e., children's use of alternative response strategies) was shown to occur on a visual localization task. We have no reason to suppose that such development does not further generalize across a wide range of other modalities and task domains. However, it should be noted that, from a practical perspective, some other domains may not be so easily studied. With 2D localization, it is straightforward and natural to think geometrically about different ways of solving the problem, for example, in terms of drawing a certain shape around a cluster of points, or mean‐averaging their locations. Furthermore, there exists an intuitive response for us to measure (pointing), which can be used to delineate between competing hypotheses. In contrast, with more complex stimuli such as faces, the decision space is hyper‐dimensional, and it becomes considerably more difficult for us—as human experimenters—to formulate the various different models that observers might employ, or to visualize/interpret the data. Meanwhile, at the other end of the spectrum, some decision spaces are so simple that explanations may “come to an end” (Wittgenstein, 2009). For example, with a one‐dimensional feature space, such as size, one can imagine a number of plausible statistics observers might use (arithmetic mean, geometric mean, median, mode, robust averages, etc.). However, there is no obvious, straightforward method of collecting the response required to test the competing hypotheses (i.e., method of adjustment is notoriously problematic; Wier, Jesteadt, & Green, 1976), and with the decision space containing only a single dimension, the observable differences in response may be too small to measure accurately in children.

## 5 SUMMARY AND CONCLUSIONS

The current study demonstrates that children's difficulties in computing summary statistics does not simply represent poor implementation of an adult‐like algorithm. Instead, children are liable to be limited further by the use of qualitatively different decision strategies—strategies which may be sensible in themselves, but not ideal given the task context. This suggests that structural learning, the ability to select the most efficient problem‐solving model for a task, is also a crucial factor in perceptual development.

## ENDNOTES

1 Adults are also capable of summarizing higher‐order perceptual features, such as the average identity (de Fockert & Wolfenstein, 2009) of a face, the heading of a crowd of people (Sweeny, Haroz, & Whitney, 2013), or the average direction of illumination in a visual scene (Koenderink, van Doorn, & Pont, 2004). They can also average non‐perceptual information, such as political approval ratings (Simonton, 1986), value for money (Levin, 1974), or moral descriptors (Leon, Oden, & Anderson, 1973).

2 Assuming that noise samples are independently random distributed (see Jones, 2016).

3 NB: this region was large enough that observers using any strategy would be predicted to receive positive feedback on the majority of trials: Minbound circ: 83.6%; Minbound triang: 92.1%; Minbound rect: 87.4%; Convex hull: 97.0%; Geometric mean: 98.9%; Fitcircle geometric: 55.6%; Fitcircle algebraic: 72.9%.

4 Additional strategies were considered, but can be discounted a priori. For example, another intuitive decision strategy is to respond to the geometric centerpoint. This is a generalization of the median, and predicts that observers responded to the dot with greatest statistical depth (e.g., Tukey halfspace depth (Tukey, 1975)). Such a strategy can be ruled out *prima facie*, however, since participants seldom pointed directly at a location containing an observed dot, and in fact were instructed explicitly that the ‘middle’ of the dot‐cloud did not necessarily contain a dot.

5 Unsurprisingly, in *N* > 6 conditions none of the age differences in response precision were explained by decision strategy, since in these conditions children and adults appeared to use the same strategy (arithmetic mean averaging).

## References

[desc12584-bib-0001] Alvarez, G.A. (2011). Representing multiple objects as an ensemble enhances visual cognition. Trends in Cognitive Sciences, 15(3), 122–131. 21292539 10.1016/j.tics.2011.01.003

[desc12584-bib-0002] Alvarez, G.A. , & Aude, O. (2008). The representation of simple ensemble visual features outside the focus of attention. Psychological Science, 19(4), 392–398. 18399893 10.1111/j.1467-9280.2008.02098.x PMC2587223

[desc12584-bib-0003] Amano, K. , Takeda, T. , Haji, T. , Terao, M. , Maruya, K. , Matsumoto, K. , & Nishida, S. (2012). Human neural responses involved in spatial pooling of locally ambiguous motion signals. Journal of Neurophysiology, 107(12), 3493–3508. 22442570 10.1152/jn.00821.2011

[desc12584-bib-0004] Ariely, D. (2001). Seeing sets: Representation by statistical properties. Psychological Science, 12(2), 157–162. 11340926 10.1111/1467-9280.00327

[desc12584-bib-0005] Attarha, M. , & Moore, C.M. (2015). The capacity limitations of orientation summary statistics. Attention, Perception, and Psychophysics, 77(4), 1116–1131. 10.3758/s13414-015-0870-0 PMC4417065 25810160

[desc12584-bib-0006] Awh, E. , Barton, B. , & Vogel, E.K. (2007). Visual working memory represents a fixed number of items regardless of complexity. Psychological Science, 18(7), 622–628. 17614871 10.1111/j.1467-9280.2007.01949.x

[desc12584-bib-0007] Badcock, D.R. , Hess, R.F. , & Dobbins, K. (1996). Localization of element clusters: Multiple cues. Vision Research, 36(10), 1467–14372. 8762763 10.1016/0042-6989(95)00205-7

[desc12584-bib-0008] Baud‐Bovy, G. , & Soechting, J. (2001). Visual localization of the center of mass of compact, asymmetric, two‐dimensional shapes. Journal of Experimental Psychology: Human Perception and Performance, 27(3), 692–706. 11424655 10.1037//0096-1523.27.3.692

[desc12584-bib-0009] Bauer, B. (2009). Does Stevens's power law for brightness extend to perceptual brightness averaging? Psychological Record, 59(2), 171–186.

[desc12584-bib-0010] Berg, B.G. (2004). A molecular description of profile analysis: Decision weights and internal noise. Journal of the Acoustical Society of America, 115(2), 822–829. 15000193 10.1121/1.1639904

[desc12584-bib-0011] Brainard, D.H. (1997). The Psychophysics Toolbox. Spatial Vision, 10(4), 433–436. 9176952

[desc12584-bib-0012] Bulatov, A. , Bertulis, A. , Gutauskas, A. , Mickiene, L. , & Kadziene, G. (2010). Center‐of‐mass alterations and visual illusion of extent. Biological Cybernetics, 102(6), 475–487. 20300941 10.1007/s00422-010-0379-5

[desc12584-bib-0013] Burr, D.C. (1981). Temporal summation of moving images by the human visual system. Proceedings of the Royal Society of London B: Biological Sciences, 211(1184), 321–339. 6111803 10.1098/rspb.1981.0010

[desc12584-bib-0014] Chong, S.C. , & Treisman, A. (2005). Statistical processing: Computing the average size in perceptual groups. Vision Research, 45(7), 891–900. 15644229 10.1016/j.visres.2004.10.004

[desc12584-bib-0015] Cowan, N. , AuBuchon, A.M. , Gilchrist, A.L. , Ricker, T.R. , & Saults, J.S. (2011). Age differences in visual working memory capacity: Not based on encoding limitations. Developmental Science, 14(5), 1066–1074. 21884322 10.1111/j.1467-7687.2011.01060.x PMC3177168

[desc12584-bib-0016] Dakin, S.C. , Bex, P.J. , Cass, J.R. , & Watt, R.J. (2009). Dissociable effects of attention and crowding on orientation averaging. Journal of Vision, 9(11), 1–16. 10.1167/9.11.28 PMC2927104 20053091

[desc12584-bib-0017] Dakin, S.C. , & Watt, R.J. (1997). The computation of orientation statistics from visual texture. Vision Research, 37(22), 3181–3192. 9463699 10.1016/s0042-6989(97)00133-8

[desc12584-bib-0018] Feller, W. (1968). An introduction to probability theory and its applications: Volume I. New York: John Wiley & Sons.

[desc12584-bib-0019] de Fockert, J. , & Wolfenstein, C. (2009). Rapid extraction of mean identity from sets of faces. Quarterly Journal of Experimental Psychology, 62(9), 1716–1722. 10.1080/17470210902811249 19382009

[desc12584-bib-0020] Gander, W. , Golub, G.H. , & Rolf Strebel, R. (1994). Least‐squares fitting of circles and ellipses. BIT Numerical Mathematics, 34(4), 558–578.

[desc12584-bib-0021] Gorea, A. , Belkoura, S. , & Solomon, J.A. (2014). Summary statistics for size over space and time. Journal of Vision, 14(9), 22. 10.1167/14.9.22 25157045

[desc12584-bib-0022] Gori, M. , Del Viva, M. , Sandini, G. , & Burr, D.C. (2008). Young children do not integrate visual and haptic form information. Current Biology, 18(9), 694–698. 18450446 10.1016/j.cub.2008.04.036

[desc12584-bib-0023] Green, D.M. (1964). Consistency of auditory detection judgments. Psychological Review, 71(5), 392–407. 14208857 10.1037/h0044520

[desc12584-bib-0024] Haberman, J. , Brady, T.F. , & Alvarez, G.A. (2015). Individual differences in ensemble perception reveal multiple, independent levels of ensemble representation. Journal of Experimental Psychology: General, 144(2), 432–446. 25844624 10.1037/xge0000053

[desc12584-bib-0025] Haberman, J. , & Whitney, D. (2012). Ensemble perception: Summarizing the scene and broadening the limits of visual processing In Wolfe J. & Robertson L. (Eds.), From perception to consciousness: Searching with Anne Treisman (pp. 339–349). Oxford: Oxford University Press.

[desc12584-bib-0026] Hess, R.F. , Dakin, S.R. , & Badcock, D. (1994). Localization of element clusters by the human visual system. Vision Research, 34(18), 2439–2451. 7975283 10.1016/0042-6989(94)90288-7

[desc12584-bib-0027] Hess, R.F. , & Holliday, I.E. (1992). The coding of spatial position by the human visual system: Effects of spatial scale and contrast. Vision Research, 32(6), 1085–1097. 1509699 10.1016/0042-6989(92)90009-8

[desc12584-bib-0028] Hubert‐Wallander, B. , & Boynton, G.M. (2015). Not all summary statistics are made equal: Evidence from extracting summaries across time. Journal of Vision, 15(4), 1–12. 10.1167/15.4.5 PMC4463776 26053144

[desc12584-bib-0029] Im, H.Y. , & Halberda, J. (2013). The effects of sampling and internal noise on the representation of ensemble average size. Attention, Perception, and Psychophysics, 75(2), 278–286. 10.3758/s13414-012-0399-4 23188732

[desc12584-bib-0030] Jacoby, O. , Kamke, M.R. , & Mattingley, J.B. (2013). Is the whole really more than the sum of its parts? Estimates of average size and orientation are susceptible to object substitution masking. Journal of Experimental Psychology: Human Perception and Performance, 39(1), 233–244. 22642216 10.1037/a0028762

[desc12584-bib-0031] Jesteadt, W. , Nizami, L. , & Schairer, K.S. (2003). A measure of internal noise based on sample discrimination. Journal of the Acoustical Society of America, 114(4), 2147–2157. 14587612 10.1121/1.1610456

[desc12584-bib-0032] Jones, P.R. (2016). A tutorial on cue combination and Signal Detection Theory: Using changes in sensitivity to evaluate how observers integrate sensory information. Journal of Mathematical Psychology, 73, 117–139.

[desc12584-bib-0033] Jones, P.R. , Moore, D.R. , & Amitay, S. (2015). Development of auditory selective attention: Why children struggle to hear in noisy environments. Developmental Psychology, 51(3), 353–369. 25706591 10.1037/a0038570 PMC4337492

[desc12584-bib-0034] Jones, P.R. , Moore, D.R. , Shub, D.E. , & Amitay, S. (2014). Learning to detect a tone in unpredictable noise. Journal of the Acoustical Society of America, 135(3), EL128–EL133. 24606305 10.1121/1.4865267

[desc12584-bib-0035] Jones, P.R. , Shub, D.E. , Moore, D.R. , & Amitay, S. (2013). Reduction of internal noise in auditory perceptual learning. Journal of the Acoustical Society of America, 133(2), 970–981. 23363114 10.1121/1.4773864

[desc12584-bib-0036] Juni, M.Z. , Gureckis, T.M. , & Maloney, L.T. (2015). Information sampling behavior with explicit sampling costs. Decision, 3(1), 147–168. 10.1037/dec0000045 PMC4942190 27429991

[desc12584-bib-0037] Koenderink, J.J. , van Doorn, A.J. , & Pont, S.C. (2004). Light direction from shad(ow)ed random Gaussian surfaces. Perception, 33(12), 1405–1420. 15729909 10.1068/p5287

[desc12584-bib-0038] Leon, M. , Oden, G.C. , & Anderson, N.H. (1973). Functional measurment of social values. Journal of Personality and Social Psychology, 27(3), 301–310.

[desc12584-bib-0039] Levin, I.P. (1974). Averaging processes in ratings and choices based on numerical information. Memory and Cognition, 2(4), 786–790. 24203755 10.3758/BF03198156

[desc12584-bib-0040] Lu, Z.L. , & Dosher, B.A. (1999). Characterizing human perceptual inefficiencies with equivalent internal noise. Journal of the Optical Society of America, 16(3), 764–778. 10069062 10.1364/josaa.16.000764

[desc12584-bib-0041] Luck, S.J. , & Vogel, E.K. (1997). The capacity of visual working memory for features and conjunctions. Nature, 390(6657), 279–281. 9384378 10.1038/36846

[desc12584-bib-0042] Lutfi, R.A. (1995). Correlation coefficients and correlation ratios as estimates of observer weights in multiple‐observation tasks. Journal of the Acoustical Society of America, 97(2), 1333–1334.

[desc12584-bib-0043] Manning, C. , Dakin, S.C. , Tibber, M.S. , & Pellicano, E. (2014). Averaging, not internal noise, limits the development of coherent motion processing. Developmental Cognitive Neuroscience, 10, 44–56. 25160679 10.1016/j.dcn.2014.07.004 PMC4256063

[desc12584-bib-0044] Marchant, A.P. , Simons, D.J. , & de Fockert, J.W. (2013). Ensemble representations: Effects of set size and item heterogeneity on average size perception. Acta Psychologica, 142(2), 245–250. 23376135 10.1016/j.actpsy.2012.11.002

[desc12584-bib-0045] Morgan, M.J. , & Glennerster, A. (1991). Efficiency of locating centres of dot‐clusters by human observers. Vision Research, 31(12), 2075–2083. 1771793 10.1016/0042-6989(91)90165-2

[desc12584-bib-0046] Morgan, M. , Chubb, C. , & Solomon, J.A. (2008). A “dipper” function for texture discrimination based on orientation variance. Journal of Vision, 8(11), 9. 10.1167/8.11.9 PMC4135071 18831603

[desc12584-bib-0047] Nardini, M. , Jones, P. , Bedford, R. , & Braddick, O. (2008). Development of cue integration in human navigation. Current Biology, 18(9), 689–693. 18450447 10.1016/j.cub.2008.04.021

[desc12584-bib-0048] Parkes, L. , Lund, J. , Angelucci, A. , Solomon, J.A. , & Morgan, M. (2001). Compulsory averaging of crowded orientation signals in human vision. Nature Neuroscience, 4(7), 739–744. 11426231 10.1038/89532

[desc12584-bib-0049] Peterson, C.R. , & Beach, L.R. (1967). Man as an intuitive statistician. Psychological Bulletin, 68(1), 29–46. 6046307 10.1037/h0024722

[desc12584-bib-0050] Petrini, K. , Jones, P.R. , Smith, L. , & Nardini, M. (2015). Hearing where the eyes see: Children use an irrelevant visual cue when localizing sounds. Child Development, 86(5), 1449–1457. 26228618 10.1111/cdev.12397

[desc12584-bib-0051] Price, P.C. , Kimura, N.M. , Smith, A.R. , & Marshall, L.D. (2014). Sample size bias in judgments of perceptual averages. Journal of Experimental Psychology: Learning, Memory, and Cognition, 40(5), 1321–1331. 10.1037/a0036576 24749965

[desc12584-bib-0052] Richards, V.M. , & Zhu, S. (1994). Relative estimates of combination weights, decision criteria, and internal noise based on correlation coefficients. Journal of the Acoustical Society of America, 95(1), 423–434. 8120253 10.1121/1.408336

[desc12584-bib-0053] Ristic, J. , & Kingstone, A. (2009). Rethinking attentional development: Reflexive and volitional orienting in children and adults. Developmental Science, 12(2), 289–296. 19143801 10.1111/j.1467-7687.2008.00756.x

[desc12584-bib-0054] Robitaille, N. , & Harris, I.M. (2011). When more is less: Extraction of summary statistics benefits from larger sets. Journal of Vision, 11(12), 18. 10.1167/11.12.18 22031908

[desc12584-bib-0055] Rocchi, F. , Ledgeway, T. , & Webb, B.S. (2013). Visual motion integration is mediated by directional ambiguities in local motion signals. Frontiers in Computational Neuroscience, 7, 167. 24302910 10.3389/fncom.2013.00167 PMC3831152

[desc12584-bib-0056] Rousseeuw, P.J. , & Croux, C. (1993). Alternatives to the median absolute deviation. Journal of the American Statistical Association, 88(424), 1273–1283.

[desc12584-bib-0057] Simmering, V.R. (2012). The development of visual working memory capacity during early childhood. Journal of Experimental Child Psychology, 111(4), 695–707. 22099167 10.1016/j.jecp.2011.10.007

[desc12584-bib-0058] Simons, D.J. , & Myczek, K. (2008). Average size perception and the allure of a new mechanism. Perception and Psychophysics, 70(7), 1335–1336. 10.3758/pp.70.5.772 18613626

[desc12584-bib-0059] Simonton, D.K. (1986). Dispositional attributions of (presidential) leadership: An experimental simulation of historiometric results. Journal of Experimental Social Psychology, 22(5), 389–418.

[desc12584-bib-0060] Snowden, R.J. , & Braddick, O.J. (1989). The combination of motion signals over time. Vision Research, 29(11), 1621–1630. 2635485 10.1016/0042-6989(89)90143-0

[desc12584-bib-0061] Solomon, J.A. , Morgan, M. , & Chubb, C. (2011). Efficiencies for the statistics of size discrimination. Journal of Vision, 11(12), 13. 10.1167/11.12.13 PMC4135075 22011381

[desc12584-bib-0062] Sweeny, T.D. , Haroz, S. , & Whitney, D. (2013). Perceiving group behavior: Sensitive ensemble coding mechanisms for biological motion of human crowds. Journal of Experimental Psychology: Human Perception and Performance, 39(2), 329–337. 22708744 10.1037/a0028712

[desc12584-bib-0063] Sweeny, T.D. , Wurnitsch, N. , Gopnik, A. , & Whitney, D. (2015). Ensemble perception of size in 4–5‐year‐old children. Developmental Science, 18(4), 556–568. 25442844 10.1111/desc.12239 PMC5282927

[desc12584-bib-0064] Swets, J.A. (1959). Multiple observations of signals in noise. Journal of the Acoustical Society of America, 31(4), 514–521.

[desc12584-bib-0065] Tukey, J.W. (1975). Mathematics and the picturing of data In Proceedings of the International Congress of Mathematicians (vol. 2, pp. 523–531). Vancouver.

[desc12584-bib-0066] Vos, P.G. , Bocheva, N. , Yakimoff, N. , & Helsper, E. (1993). Perceived location of two‐dimensional patterns. Vision Research, 33(15), 2157–2169. 8266657 10.1016/0042-6989(93)90014-n

[desc12584-bib-0067] Ward, R. , Casco, C. , & Watt, R.J. (1985). The location of noisy visual stimuli. Canadian Journal of Psychology/Revue Canadienne de Psychologie, 39(3), 387–399. 10.1037/h0080067 4052880

[desc12584-bib-0068] Watamaniuk, S.N.J. , & Duchon, A. (1992). The human visual system averages speed information. Vision Research, 32(5), 931–941. 1604862 10.1016/0042-6989(92)90036-i

[desc12584-bib-0069] Watamaniuk, S.N.J. , Sekuler, R. , & Williams, D.W. (1989). Direction perception in complex dynamic displays: The integration of direction information. Vision Research, 29(1), 47–59. 2773336 10.1016/0042-6989(89)90173-9

[desc12584-bib-0070] Whelan, R. (2008). Effective analysis of reaction time data. Psychological Record, 58(3), 475–482.

[desc12584-bib-0071] Whitaker, D. , McGraw, P.V. , Pacey, I. , & Barrett, B.T. (1996). Centroid analysis predicts visual localization of first‐ and second‐order stimuli. Vision Research, 36(18), 2957–2970. 8917796 10.1016/0042-6989(96)00031-4

[desc12584-bib-0072] Whitaker, D. , & Walker, H. (1988). Centroid evaluation in the vernier alignment of random dot clusters. Vision Research, 28(7), 777–784. 3227654 10.1016/0042-6989(88)90024-7

[desc12584-bib-0073] Wier, C.C. , Jesteadt, W. , & Green, D.M. (1976). A comparison of method‐of‐adjustment and forced‐choice procedures in frequency discrimination. Attention, Perception, and Psychophysics, 19(1), 75–79.

[desc12584-bib-0074] Williams, D.W. , & Sekuler, R. (1984). Coherent global motion percepts from stochastic local motions. Vision Research, 24(1), 55–62. 6695508 10.1016/0042-6989(84)90144-5

[desc12584-bib-0075] Wittgenstein, L. (2009). Philosophical Investigations (Anscombe G. E. M., Hacker P. M. S., Schulte Trans J.). New York: John Wiley & Sons. (Original work published 1953).

[desc12584-bib-0076] Wolpert, D.M. , & Flanagan, J.R. (2010). Motor learning. Current Biology, 20(11), R467–R472. 20541489 10.1016/j.cub.2010.04.035

